# Endothelial disruption: A new complication during pulsed field ablation using a pentaspline catheter

**DOI:** 10.1016/j.hrcr.2026.01.019

**Published:** 2026-01-30

**Authors:** Ji Mei May Wong, Ali R. Keramati, Elliot Brown, Bruce Skolnick, Suneet Mittal, Mohammadali Habibi

**Affiliations:** 1 Department of Internal Medicine at Lankenau Medical Center, Wynnewood, Pennsylvania; 2 Department of Cardiology, Lankenau Heart Institute, Wynnewood, Pennsylvania; 3 Department of Cardiology, Valley Health System, Paramus, New Jersey

**Keywords:** Endothelial disruption, Pulsed field ablation, Intracardiac echocardiography, Atrial fibrillation, FARAPULSE system

**Key Teaching Points**

• Endothelial disruption is a subtle but potentially clinically significant complication of pulsed field ablation that may result from either direct electroporation-related injury or catheter-induced mechanical trauma.
• Even minor endothelial injury in low-flow regions of the left atrium, such as the roof or appendage, can predispose to thrombus formation and may contribute to cerebrovascular complications during or after pulsed field ablation procedures.
• When positioned in the left atrium, intracardiac echocardiography can detect areas of endothelial disruption that may be missed with imaging from the right atrium.

## Introduction

Pulsed field ablation (PFA) is an emerging nonthermal ablation technique that is increasingly being used for the treatment of atrial fibrillation (AF). It works by delivering rapid intermittent high-voltage pulses to myocardial tissue, inducing irreversible electroporation—a process that increases membrane permeability, disrupts cellular homeostasis, and promotes death of affected cells via apoptosis, necrosis, necroptosis, and pyroptosis.^1^^,^^2^ PFA has been shown to be safe and effective in achieving pulmonary vein isolation (PVI), while sparing damage to collateral structures such as the esophagus, phrenic nerve, and distal pulmonary veins—injuries more classically associated with traditional thermal ablation techniques.^3^ Nevertheless, PFA may not be without other risks. We present 2 cases of endothelial disruption observed during PFA, which were detected with intracardiac echocardiography (ICE) positioned in the left atrium (LA).

## Case report

Patient #1 is a 62-year-old man with hypertension who was diagnosed as having paroxysmal AF 6 months before referral to electrophysiology. During this period, his AF episodes increased in duration and frequency, eventually becoming persistent with accompanying symptoms of palpitations and fatigue. He was started on apixaban 5 mg twice daily and nebivolol 5 mg daily for rate control; other medications included candesartan for hypertension. Transthoracic echocardiography showed a normal left ventricular ejection fraction of 60% and mild LA dilatation (LA volume index 38 mL/m^2^) without significant valvular or structural heart disease.

Given his relatively young age and progression to persistent AF, rhythm-control strategies—including cardioversion, antiarrhythmic drug therapy, and catheter ablation—were discussed. The patient opted for PFA, which was performed using a 31 mm pentaspline catheter (FARAPULSE system, Boston Scientific). Anticoagulation leading up to the procedure was uninterrupted, with the last apixaban dose taken the morning of the procedure. Baseline ICE images from the right atrium (RA) showed no evidence of an intracardiac thrombus. An intravenous heparin bolus followed by heparin infusion was given throughout the procedure to maintain an activated clotting time (ACT) of >300 seconds. Serial ACTs during the procedure measured 358, 363, 348, and 308 seconds. The procedure was performed using zero fluoroscopy. Single transseptal access was obtained under ICE guidance using a VersaCross wire (Boston Scientific). A deflectable 13F sheath (FaraDrive, Boston Scientific) was advanced into the LA under ICE guidance. Via the same access, an ICE catheter was positioned in the LA to optimize assessment of ablation catheter-tissue contact. Again, there was no evidence of an intracardiac thrombus. 3-dimensional electroanatomic mapping of the LA was performed using the pentaspline catheter with the ESI-X/Abbott mapping system. PVI was performed using the PFA catheter in both “basket” and “flower” configurations. The posterior wall was ablated using a series of PFA applications in the “flower” configuration. No increase in esophageal temperature, as measured by a probe inserted by the anesthesia team for core body temperature monitoring, was observed during PFA applications. The procedure involved a total of 58 PFA applications.

After ablation, a clear interruption within the LA endothelium along the posterior wall was noted, with 2 attached mobile masses each measuring approximately 1.2 cm in length (Supplemental Video 1). These mobile structures were not present at the start of the case nor during ablation. In the same ICE clip (Supplemental Video 1), 1 fragment of the endothelial tissue appears to detach from the LA and embolize. Given the unclear nature of these structures and the possibility of concomitant thrombus formation, cardioversion was not performed. The catheters were then removed from the LA. Interestingly, the mobile structures became barely visible with repositioning of the ICE catheter to the RA (Supplemental Video 2). A 3-dimensional transesophageal echocardiogram (TEE) was obtained for further assessment of the mobile masses and to establish a baseline for future monitoring (Supplemental Video 3). The pedunculated mobile structures attached to the posterior wall of the LA were redemonstrated.

After the procedure, the patient had no neurologic symptoms and had no deficits noted on physical examination such that magnetic resonance imaging (MRI) of the brain was not obtained. He was discharged on the same day to continue on his home apixaban and nebivolol. He returned for a follow-up visit after having completed 5 weeks of therapeutic anticoagulation and reported that he was mostly asymptomatic except for occasional palpitations. Repeat TEE revealed complete resolution of the endothelial lesions with no evidence of an intra-atrial thrombus, and thus, direct current cardioversion was performed.

Patient #2 is a 70-year-old man with chronic diastolic heart failure with a preserved ejection fraction of 50%–55%, hypertension, and a recent nuclear stress test with no evidence of ischemia, who was diagnosed as having paroxysmal AF 8 months before evaluation by the electrophysiology service. During this period, his AF was refractory to both rate control with metoprolol succinate and electrical cardioversion. He was eventually started on rivaroxaban 20 mg daily, metoprolol succinate 100 mg daily, and diltiazem 120 mg daily for rate control. Amiodarone 200 mg daily (after a taper) was started for rhythm control. Other regular medications included losartan for hypertension.

The patient presented to the emergency department with acute decompensated heart failure, attributed to AF with rapid ventricular response. During this hospitalization, he was treated with intravenous bumetanide for volume overload and placed on bilevel positive airway pressure for respiratory support. Transthoracic echocardiography showed a normal left ventricular ejection fraction of 50%–55%, a normally sized LA (LA volume index 20 mL/m^2^), and mild tricuspid and mitral regurgitation. TEE identified no thrombus or mass in the LA or LA appendage. The patient underwent an electrical cardioversion with initial restoration of sinus rhythm, although frequent premature atrial contractions were noted. He reverted to AF with rapid ventricular response within hours.

Owing to persistent AF, a decision was made to pursue catheter ablation with a 31 mm pentaspline catheter (FARAPULSE system, Boston Scientific). Anticoagulation leading up to the procedure was uninterrupted, with the last rivaroxaban dose taken the day of the procedure. Baseline ICE showed no evidence of an intracardiac thrombus. A heparin bolus was administered at the start of the procedure, which was then maintained with a continuous heparin infusion. Single transseptal access was obtained using a VersaCross wire under ICE guidance. Via this access, both a deflectable 13F sheath (Boston Scientific) and an ICE catheter were advanced into the LA. Serial ACTs after transseptal puncture measured 331 and 302 seconds. The patient was externally cardioverted to normal sinus rhythm. 3-dimensional electroanatomic mapping of the LA was performed during sinus rhythm using the pentaspline catheter with the ESI-X/Abbott mapping system. This revealed a significant amount of scar tissue and fractionated signals over the posterior wall of the LA. PVI was conducted using the PFA catheter in both “basket” and “flower” configurations. Owing to significant scarring of the posterior wall, a decision was made to proceed with posterior wall isolation. Complete posterior wall isolation was achieved with a series of PFA applications using the “flower” configuration along the LA roof and floor lines. The procedure involved a total of 46 PFA applications.

As the case concluded, a 1 cm lesion on the LA roof was noted on ICE (Supplemental Video 4). The lesion did not appear to be attached to the ablation catheter. The catheter was withdrawn from the LA, and the procedure was terminated. The patient was immediately started on treatment-dose enoxaparin postoperatively for 2 doses, before being transitioned to apixaban. After the procedure, the patient exhibited no neurologic deficits. Guideline-directed medical therapy was optimized before discharge. The patient had an uneventful recovery and was discharged home. He remained in normal sinus rhythm at his 3-week follow-up visit.

## Discussion

We describe 2 cases of LA endothelial disruption during PFA that, although subtle, may be of clinical significance. We hypothesize that this occurs during PFA primarily via 2 mechanisms: (1) the direct effects of PFA application on endothelial tissue and/or (2) damaging or scratching of endothelial tissue secondary to catheter-related mechanical trauma.

Although there remains a paucity of literature clearly linking the effects of irreversible electroporation in PFA with endothelial disruption, studies suggest several possible mechanisms. First, the increased membrane permeability from electroporation can cause calcium influx into affected cells, which triggers contraction and cortical cytoskeleton disassembly.^4^ This may result in loss of cellular shape and intercellular adhesions, which may predispose to endothelial disruption—likely reflecting localized denudation. Second, although PFA is generally thought to be a nonthermal ablation technique, repetitive high-voltage pulses will inevitably produce some local heating^5^; in other words, cells closest to the catheter electrode may sustain thermal injury, presenting another pathway in which endothelial disruption may occur. However, in 1 case in which an esophageal temperature probe was placed, no increase in esophageal temperature was observed. Finally, PFA has been associated with coronary artery vasospasm, likely mediated by electrical pulse–induced neuron depolarization.^5^^,^^6^ By extension, such depolarization may also provoke vasoconstriction of local blood vessels, with transient reductions in microvasculature perfusion possibly contributing to endothelial disruption.

Catheter-related mechanical trauma is another plausible explanation. Although the unique shape-shifting apparatus of the FARAPULSE system (Boston Scientific) is useful in accommodating anatomic variation, it creates a relatively bulky distal profile that—when fully expanded—can scratch the LA wall and cause accidental endothelial disruption. During ablation procedures for AF, the catheter must be manipulated into complex configurations to achieve adequate spline-tissue apposition, especially when targeting the posterior wall of the LA. Although necessary for achieving complete lesion coverage, these maneuvers can result in unintended contact between the PFA catheter and the LA endocardium beyond the pulmonary veins. Repeated repositioning, torquing, or pressure from the catheter splines may strip the endothelial lining, exposing subendothelial tissue. Because of this, the risk of endothelial disruption may be heightened in anatomically challenging cases or in redo procedures.

Alternative etiologies, such as intracardiac thrombi, were also considered but deemed less likely given uninterrupted anticoagulation leading up to and after the procedure and therapeutic intraprocedural ACTs. On echocardiography, these mass-like lesions were observed to be linear and serpentine in nature with a clearly demonstrated point of attachment to the endocardium. In contrast, thrombi are characteristically heterogeneous, irregular, echodense materials with a more globular morphology, features absent in our cases. In addition, these lesions were noted to be in locations atypical for thrombi formation, which more commonly occurs in the left ventricular apex, LA appendage, and isolated segments of ventricular akinesis or dyskinesis.

**Figure 1:**
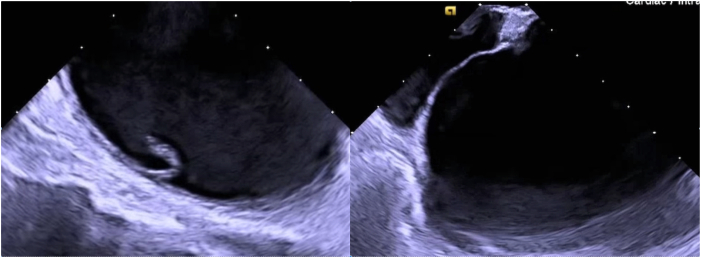
Endothelial disruption of the left atrium after pulsed field ablation. The lesion is clearly visible with intracardiac echocardiography placed inside the left atrium (left), but not clearly when images were obtained from the right atrium (right).

Interestingly, the areas of endothelial disruption noted in our 2 cases were only appreciable with the ICE catheter placed in the LA; retraction of the ICE catheter into the RA resulted in loss of clear visualization of these small pedunculated lesions (Figure 1). This likely reflects the enhanced near-field resolution, improved ICE catheter manipulation (to achieve perpendicular insonation), and inherent proximity to the LA walls when performing ICE from the LA vs the RA. Because of how easily it can go undetected, endothelial disruption may be more common than appreciated. It is also important to note that both reported cases involved using the FARAPULSE system (Boston Scientific) and pentaspline catheters; therefore, these findings may not be generalizable to other PFA systems or catheters. The short- and long-term consequences of LA endothelial disruption remain uncertain; in our 2 cases, no immediate- or short-term adverse events were identified. Nevertheless, exposure of the subendothelial matrix is highly thrombogenic and may promote thrombus formation with downstream cerebrovascular risk. Although our patients did not exhibit any neurologic deficits after PFA, embolization of endothelial tissue can result in silent cerebral events, complications that have been reported after ablation procedures for AF.^3^^,^^7^ Unfortunately, a key limitation of our case series is the absence of a brain MRI to screen for subclinical cerebrovascular events. Still, detection of endothelial disruption highlights the importance of appropriate anticoagulation during and after the ablation procedure to reduce the risk of embolic events.

## Conclusions

Although PFA largely avoids many of the collateral injuries associated with traditional thermal ablation techniques including atrioesophageal fistulae, phrenic nerve palsy, and pulmonary vein stenosis, endothelial disruption—whether from the direct effects of irreversible electroporation or from catheter manipulation—remains a potentially important yet likely underdetected finding. These observations are limited by a small sample size and a lack of histologic confirmation, but suggest that ICE-guided assessment from the LA and prospective tracking of thrombotic sequelae could help clarify incidence and risk factors to guide best practice moving forward.

## Disclosures

Suneet Mittal disclosed that he is a consultant to Boston Scientific and Medtronic. Otherwise, they have no additional relationships to disclose, no patents to declare, and no other activities to report.

## Footnotes

Appendix Supplementary data associated with this article can be found in the online version at https://doi.org/10.1016/j.hrcr.2026.01.019.
