# Supplementary material for: Endothelial disruption: A new complication during pulsed field ablation using a pentaspline catheter
Source: HeartRhythm Case Rep. 2026 Jan 30;12(7):689–92. doi: 10.1016/j.hrcr.2026.01.019 (PMC13379350; doi:10.1016/j.hrcr.2026.01.019)
Supplement: Supplementary Material [file mmc1.docx]

**Video legends**

**Video 1.** Intracardiac echocardiography view of the posterior left atrial wall with the catheter positioned within the left atrium, showing disruption of the left atrial endothelium.

**Video 2.** Intracardiac echocardiography view of the posterior left atrial wall with the catheter positioned within the right atrium. Disrupted endothelium is not clearly visualized and can be easily missed.

**Video 3.** Three-dimensional reconstruction of the disrupted endothelial tissue using transesophageal echocardiography.

**Video 4.** Intracardiac echocardiography view of the posterior left atrial wall with the catheter positioned within the left atrium, showing disruption of the left atrial endothelium.
